# Low-cost, open-source device for simultaneously subjecting rodents to different circadian cycles of light, food, and temperature

**DOI:** 10.3389/fphys.2024.1356787

**Published:** 2024-02-16

**Authors:** Ramon Farré, Miguel A. Rodríguez-Lázaro, Jorge Otero, Núria Gavara, Raimon Sunyer, Núria Farré, David Gozal, Isaac Almendros

**Affiliations:** ^1^ Unit of Biophysics and Bioengineering, School of Medicine and Health Sciences, University of Barcelona, Barcelona, Spain; ^2^ CIBER de Enfermedades Respiratorias (CIBERES), Barcelona, Spain; ^3^ Institut Investigacions Biomèdiques August Pi Sunyer (IDIBAPS), Barcelona, Spain; ^4^ The Institute for Bioengineering of Catalonia (IBEC), The Barcelona Institute of Science and Technology (BIST), Barcelona, Spain; ^5^ CIBER de Bioingeniería, Biomateriales y Nanomedicina (CIBER-BBN), Barcelona, Spain; ^6^ Discipline of Cardiology, Saolta University Healthcare Group, Galway, Ireland; ^7^ School of Medicine, University of Galway, Galway, Ireland; ^8^ Office of the Dean, Joan C. Edwards School of Medicine, Marshall University, Huntington, WV, United States

**Keywords:** circadian alteration, light cycle, intermittent fasting, temperature cycle, animal research, experimental model, open-source hardware

## Abstract

Exposure of experimental rodents to controlled cycles of light, food, and temperature is important when investigating alterations in circadian cycles that profoundly influence health and disease. However, applying such stimuli simultaneously is difficult in practice. We aimed to design, build, test, and open-source describe a simple device that subjects a conventional mouse cage to independent cycles of physiologically relevant environmental variables. The device is based on a box enclosing the rodent cage to modify the light, feeding, and temperature environments. The device provides temperature-controlled air conditioning (heating or cooling) by a Peltier module and includes programmable feeding and illumination. All functions are set by a user-friendly front panel for independent cycle programming. Bench testing with a model simulating the CO_2_ production of mice in the cage showed: a) suitable air renewal (by measuring actual ambient CO_2_), b) controlled realistic illumination at the mouse enclosure (measured by a photometer), c) stable temperature control, and d) correct cycling of light, feeding, and temperature. The cost of all the supplies (retail purchased by e-commerce) was <300 US$. Detailed technical information is open-source provided, allowing for any user to reliably reproduce or modify the device. This approach can considerably facilitate circadian research since using one of the described low-cost devices for any mouse group with a given light-food-temperature paradigm allows for all the experiments to be performed simultaneously, thereby requiring no changes in the light/temperature of a general-use laboratory.

## 1 Introduction

Like all mammals and most organisms, the human body experiences the influence of the 24-h day/night cycle caused by Earth’s rotation (Stanton et al., 2022). This circadian cycle entails periodic changes in ambient light and temperature and modulates the temporal pattern of sleep and food ingestion (Fishbein et al., 2021). Homeostasis is perfectly adapted to such environmental oscillations by a system of circadian regulatory genes present not only in neurons of the suprachiasmatic nucleus but in virtually all peripheral cells (Fagiani et al., 2022). Consequently, alterations in the ambient circadian cycle perturb the expression of circadian genes and thus homeostasis (Xie et al., 2019), potentially causing or aggravating a wide range of end-organ dysfunction, e.g., metabolic (Gutierrez Lopez et al., 2021), insulin resistance (Stenvers et al., 2019), neurocognitive (Yalçin et al., 2022), vascular (Han et al., 2021), cardiac (Delisle et al., 2021), renal (Mohandas et al., 2022), fertility (Sciarra et al., 2020), or cancer (Qu et al., 2023).

Most research on the mechanisms determining the physiological adaptation to the circadian cycle, and the disruptions induced by circadian alterations, is carried out in animals, mainly in rodents, using relatively simple experimental settings. Indeed, modifying the timing of lab room illumination mimics alterations in the light/dark cycle, timed feeders can impose changes in food ingestion patterns, and setting the programmable thermostat in the lab room air-conditioning allows for modifying the ambient temperature cycles. However, performing experiments that better mimic real life by simultaneously combining alterations in the cycles of light, food ingestion, and ambient temperature is difficult given the common logistic and practical issues that arise from such experiments.

Specifically, studies comparing the impact of different combinations of light and temperature patterns would require changing the ambient conditions of the lab room for each group of mice being subjected to a given specific ambient paradigm. This would make it impossible to carry out the experiments in all the different groups simultaneously and would prevent using the animal facility room for other concurrent mouse experiments under conventional light and temperature conditions. Therefore, we aimed to design a device that would apply to any single rodent cage to create a specific ambient paradigm (regarding light, feed, and temperature cycling) so that different circadian conditions can be applied simultaneously to several animal groups within any single laboratory room. Notably, the solution we provide is simple, low-cost, easily scalable and, since we present it in an open-source format (Farré et al., 2022a; Farré et al., 2022b), it is freely adaptable by any interested research team.

## 2 Methods

### 2.1 Device description

**FIGURE 1:**
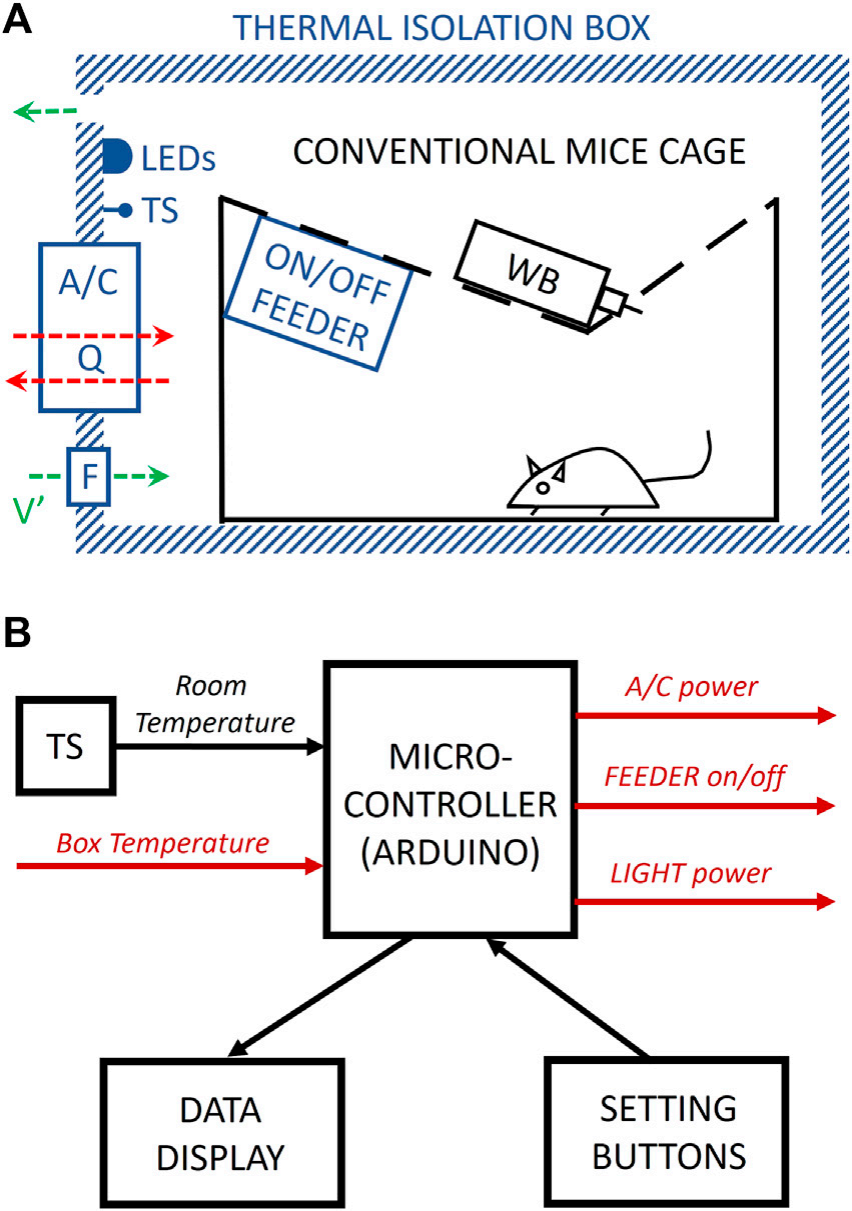
Diagram of the device. **(A)** A conventional mouse cage with a water bottle (WB) is enclosed inside an opaque and thermal isolation box. Warm-white LEDs and a temperature sensor (TS) are placed inside the box. An air-conditioning (A/C) Peltier-based module is placed through the box wall, allowing heat (Q) to flow (red arrows) from the box to the room and *vice versa* when acting as a cooler or heater, respectively. An on/off feeder allows for controlling food availability. A fan and an orifice in the box walls allow continuous airflow (V′) for air renewal (green arrows). **(B)** A microcontroller drives the cycling of A/C, feeder, and light based on the box and the room temperature (measured by a temperature sensor (TS)) and according to the settings established by the user. Signals from/to the box in red color. See the text for a detailed explanation.

Figure 1A shows a general schematic of the box. The specific implementation we present herein can accommodate either a conventional mouse cage (28 cm × 28 cm × 16 cm) or a cage (40.6 cm × 26.7 cm × 36.8 cm) for subjecting mice to sleep fragmentation/deprivation (model 80391, Lafayette Instruments, Lafayette, IN) (Cabrera-Aguilera et al., 2020; Farré et al., 2022b). To this end, we used an expanded polystyrene foam box (external 59.5 cm × 39.5 cm × 40 cm, 3 cm wall width; Broxon GmbH via Amazon) commercialized for food temperature maintenance. A 4-cm diameter personal computer fan (FAN4, AABCOOLING, via Amazon) continuously circulates room air through the box for air renewal.

A circuit (Figure 1B), based on an Arduino Nano microcontroller (Arduino, via Amazon) and a real-time clock unit (DS1307 RTC I2C, Fasici, via Amazon), is placed into a 3D-printed PLA enclosure box to control the device. The box’s front panel has displays and buttons allowing the user to independently set the cycling times for light and feeding and the temperatures and times for the thermal cycles (see details in Supplementary Materials).

**FIGURE 2:**
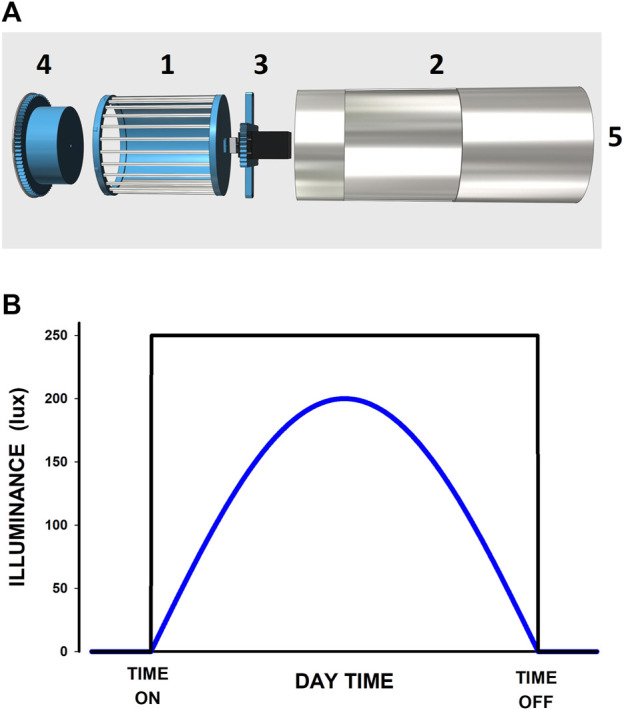
**(A)** Diagram of unmounted pieces of the on/off feeder. 1: internal cylinder to contain the conventional mice chop. One lateral part is made with stainless steel bars and the other lateral part is 3D-printed PLA (internal) and stainless steel (external, not seen in the figure). 2: External cylinder with a window. 3: Piece to be connected to the internal cylinder. 4: 3D-printed cap. 5: the place for a rotating motor. When the pieces are mounted (by displacing them from left to right), the internal cylinder can rotate inside the external one, allowing the mice (in front of the window of the external cylinder) to access the chop when the internal cylinder presents its lateral side with bars or preventing chop access when presenting the stainless-steel wall. **(B)** Two examples of the selectable illumination patterns applied to mice. The user can select whether the light pattern is constant (black) or progressive thus mimicking natural solar light (blue). The daytime of light on and off, and the illuminance amplitude in lux (250 and 200 lux, respectively, in the examples) can also be set by the user.

A customized feeder to control food availability for mice was built using cheap, commonly available, easy-to-mechanize materials (Figure 2A). All the surfaces potentially in contact with mice are made from stainless steel. We cut the external cylinder (81-mm diameter, 170-mm height) from a stainless-steel beverage bottle (1-L, Tripple Tree via Amazon), and we made the internal cage for containing conventional mice chow by using 3D-printed polylactic acid (PLA) pieces (covered with 0.1-mm width stainless steel foil where required) and stainless-steel bars (2.5-mm diameter). Mice have access to food depending on the relative position of the internal cage vs the external windowed cylinder. Rotating the internal cage with a servomotor (commercialized for aeromodelling) (HS-325HB, HITEC, via Amazon) changes the on/off feeding state.

Fifteen LED units (12 V, warm-white 3000 K; Lighting EVER via Amazon) allow illumination into the mouse cage. To set an adequate illuminance level at the mouse location, we placed the sensing element of a photometer (DM-lx1010bsxxx, Dr. Meter via Amazon) at the bottom of the mouse cage when the box was closed in working conditions. When the LEDs were continuously powered with the 12 V tension, the local il-luminance was 370 lux at the center of the mice box bottom, which is in keeping with a well-illuminated animal research lab (Dauchy and Blask, 2023). However, any illuminance value below this figure can be achieved by further reducing the duty cycle of the 12-V pulse-width modulated (PWM) powering the LEDs as set in the front panel of the device (see Supplementary Material for user manual). The device allows selecting one of two different time patterns of illumination: a simple on/off or a half-wave rectified sine simulating the progressive increase/decrease of intensity along the day (Oteiza and Pérez-Burgos, 2012) (Figure 2B).

A Peltier cooling module, typically commercialized for cooling microprocessors inside computers (120 W, 12 V, TEC1-12706, YWBL-WH, via Amazon), is placed through the box wall, with the cooling and heating sides (both including fans continuously working regardless of heating/cooling) inside and outside the box, respectively. The A/C fans in the internal side of the box contribute to homogenizing the gas composition by mixing air renewal flow and mice breathing gases). When a nominal positive voltage of 12 V powers the Peltier module, it reduces the temperature inside the box. However, when this A/C unit is powered with a negative voltage, the heat flow is inverted, and the box is heated. The polarity of the power applied to the Peltier module, and thus whether it works to cool or heat the box, is determined by the microcontroller depending on whether the room temperature sensed by a thermometer (SHT21 HTU21, HiLetgo, via Amazon) is higher or lower than the setting for box temperature, respectively. The microprocessor uses another thermometer (SHTC3, Youmile, via Amazon) inside the box to control the on/off operation of the A/C unit.

### 2.2 Performance test

We assessed the device’s performance under real-life conditions in well-controlled bench conditions freed from biological variability. The most relevant issue to test was the interaction between box temperature and air renewal flow (V′). Indeed, each of these variables conflicts with the other since excessively increasing V′ may compromise the capacity of the A/C system to control temperature. On the contrary, a too-poor air renewal may excessively increase CO_2_ inside the mouse cage. Therefore, we realistically mimicked the CO_2_ produced by five mice (a typical number for the cage employed) by introducing a CO_2_ flow of 10 mL·min^-1^ at the mouse cage bottom using narrow tubing and a servo-controlled gas blender (McQ, Virginia, United States). We established this value by assuming 30-g mice and that CO_2_ production by mouse metabolism is 4 l_CO2_·(h·g)^−1^ (Kim and Tong, 2017). According to conventional regulations (American Society of Heating, 2022), we aimed to limit any increase in the box CO_2_ concentration to 500 ppm (i.e., 5 × 10^−4^ ml_CO2_·ml_air_^−1^) above the room CO_2_ level (which means increasing the CO_2_ fraction in the air by 0.05%). To test the device performance under such realistic conditions, we measured the actual CO_2_ concentrations both in the room air and inside the cage using a commercially available CO_2_ sensor (Dioxcare DX700 PDF, Girona, Spain).

### 2.3 Construction details

All the components used to build the device were retail purchased via e-commerce (Amazon or Alibaba). The total cost of the device components, including the microprocessor, electrical and electronic components, power source, box, and mechanical parts, was <300 € (<300 US$). As the design is modular and all components are commercialized for general-purpose use, they are 9 easy to find and to be replaced by others with similar features if required. The construction is robust and its routine operation does not require the use of consumables. The energy consumption is low on a daily average (mainly depending on the lowest temperature set), with a peak of just 150 W during the phases of maximum cooling.

Full details of the electronic components, circuits and connections, mechanical pieces and their assembly, 3D-printer codes for parts and for the enclosure box, the Arduino control code, and a user manual, are freely undisclosed and available in the Supplementary Materials so that any interested user can replicate the device or adapt it to specific needs.

## 3 Results

**FIGURE 3:**
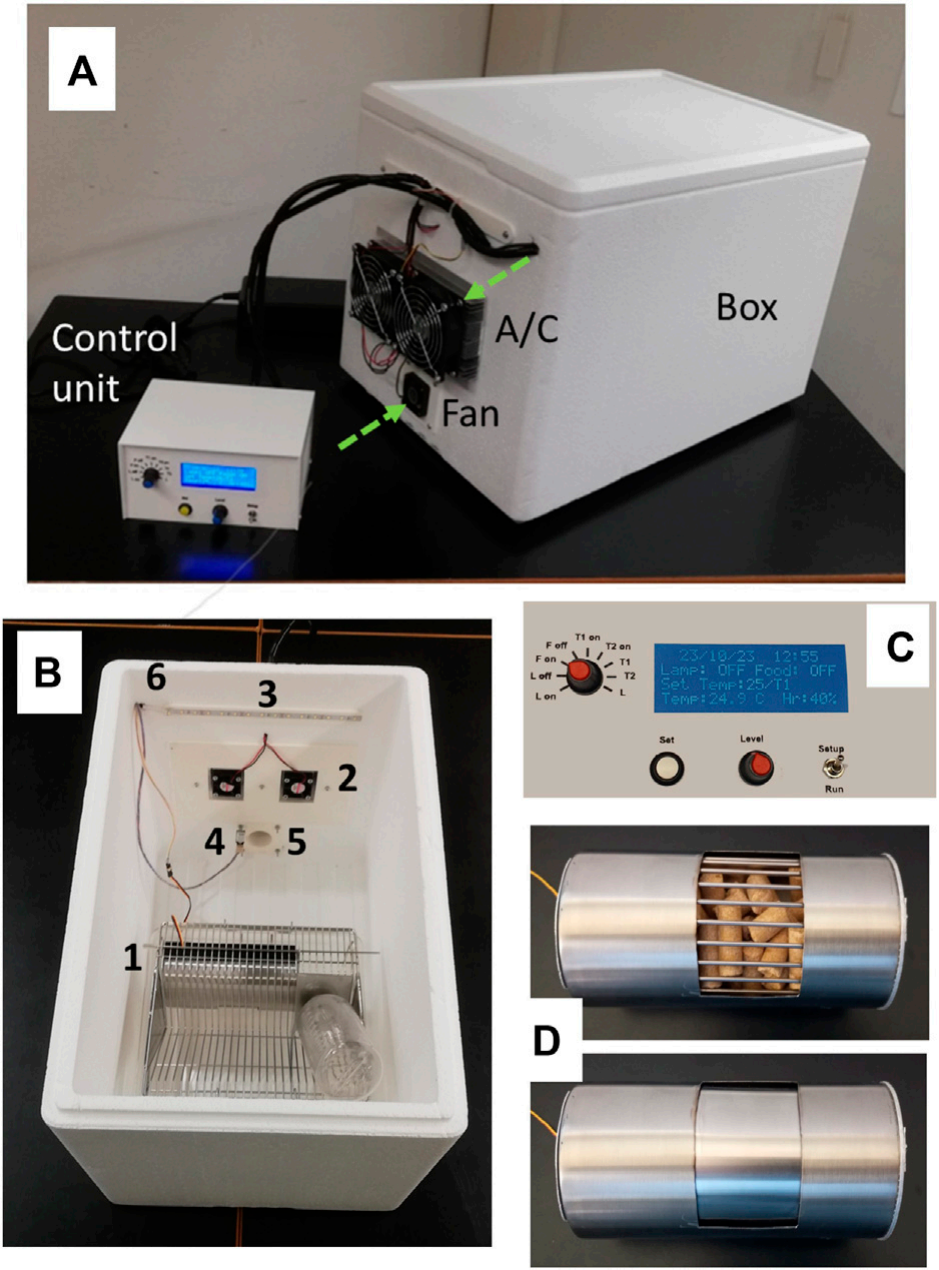
**(A)** General view of the setting, showing the box to enclose the mouse cage, the control unit, the external part of the Peltier-based air conditioning (A/C) unit, the fan for introducing room air into the box, and the wall orifice used for passing the electrical lines that also serves as the outlet of the air renewal system (green arrows represent the airflow). **(B)** Internal top view of the box showing the conventional mouse cage incorporating the on/off feeder (1), the internal blowers of the air conditioning unit (2), the illumination LEDs (3), the temperature sensor (4), the entrance for the air renewal from the fan (placed at the external side of the wall) (5), and the orifice used for passing electrical lines and used as the air outlet (6). **(C)** Detail of the front panel of the control unit. **(D)** Feeder perspective as seen by the mice, with the window open (top) and closed (bottom) making food available and unavailable to them, respectively.

A general perspective of the specific box implemented to enclose the rodent cage and modify its environment and the control unit to set the cycling parameters are both shown in Figure 3A. The material of the box walls is opaque to isolate the animals from external light and with low thermal conductivity to facilitate temperature control. Figure 3B also shows the box open to present the components within the device. Illumination inside the mouse cage box is provided by intensity-controlled light-emitting diodes (LEDs), a customized on/off feeder is placed inside the mouse cage to control food availability, and a simple air-conditioning (A/C) unit allows to modulate the temperature within the box (either below or above room temperature). The air inside the box is renewed by a fan and an exhaust orifice (Figure 3A). Figure 3C shows the front panel of the control unit which allows the user to set and monitor the cycling variables.

Figure 2B shows examples of the on/off and progressive patterns of illumination at the bottom of the mouse cage. The illuminance across different points in the bottom of the mouse cage was uniform with small variations (±30) lux depending on the exact position. It should be mentioned that the 3-cm polystyrene foam wall of the box is not completely opaque. For instance, when the closed box was placed in a well-illuminated room (≈600 lux) and the LEDs switched off), the illuminance at the mice site was ≈15 lux. In the case of aiming for a completely darker nocturnal period, the box walls can simply be painted black or lined with an opaque plastic/cardboard sheet. For instance, when the box in a well 600-lux illuminated room was covered with a black cloth cover, the illuminance measured inside the mouse cage was, as expected, zero lux (i.e., below the 1-lux resolution).

Photographs of the on-off feeder built to control food availability when placed inside a conventional mouse cage, and a detailed view of the feeder (from the mouse perspective), when food is available and unavailable, are shown in Figure 3D. The transition time between both positions is 30 s to minimally disturb the animals.

**FIGURE 4:**
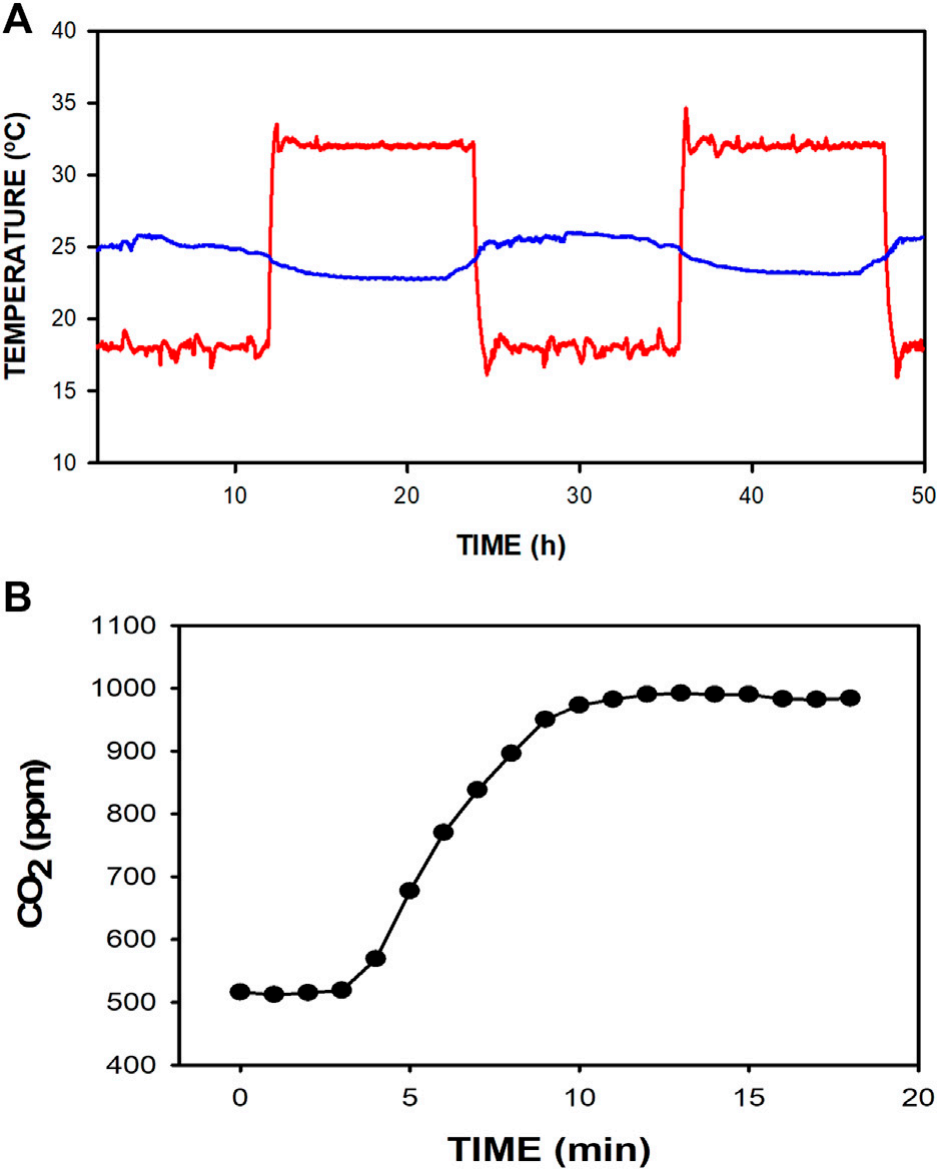
**(A)** Example of raw temperatures recorded in the box (red line) and the room (blue line) when the device was normally working for 48 h for box temperature set to cycle between 18°C and 32°C. **(B)** Change in CO_2_ concentration inside the box when a continuous flow of CO_2_ mimicking the production by 5 mice (10 mL·min^-1^) was injected into the mouse cage at min 3. CO_2_ concentration increases by less than the safe threshold of 500 ppm above the room air value.

Performance testing showed that the A/C system allows the application of controlled temperature cycling inside the box. The temperature could effectively be cycled within ±10°C around the room temperature regardless of the normal oscillations of the lab room temperature, as shown in Figure 4A when the temperature was set to cycle between 18°C and 32°C. Of note, completely opening the box by retiring its lid for 1 min (e.g., for manipulating the mice cage) induced box temperature changes lower than 1°C. Moreover, under working conditions, when 10 mL min^−1^ of CO_2_ mimicking mice production was introduced into the mouse cage, the measured CO_2_ concentration inside the box increased by only less than 500 ppm above that of the lab ambient air. Figure 4B shows that at times 0–3 min, box CO_2_ concentration was the same as in the room air (≈510 ppm) since the fan injected room air into the box. However, at time 3 min, a continuous flow of 10 mL min^−1^ of CO_2_ was injected into the mouse cage simulating the amount of CO_2_ produced by 5 mice. As expected, CO_2_ concentration started to increase until achieving a steady state of only ≈990 ppm after approximately 8 min. This recording shows that the renewal airflow through the box kept the increase in CO_2_ concentration within a safe threshold (<500 ppm) above the reference room air value.

## 4 Discussion

In this work, we have designed, built, bench-tested, and open-source described a simple device that enables a conventional mouse cage to deliver independent cycles of physiologically relevant environmental variables. This approach can considerably facilitate integrative physiology research regarding ambient variables and the circadian cycle. Indeed, using one of the low-cost devices described here for any group of mice with a given light-food-temperature paradigm could allow performing all the experiments simultaneously with no changes required in the light/temperature of the room lab, hence allowing it to be used concurrently for mouse experiments under conventional ambient conditions. Remarkably, only general-purpose and cheap components available via e-commerce are employed, and the device construction needs only simple mechanical and electronic tools, requiring technical training not higher than the level of a bachelor in engineering.

Although we could have used any commercially available programmable feeder in our setting, we designed and built a specific one from simple materials to reduce the cost. Our solution has all parts accessible to mice made of stainless steel to prevent them from gnawing, and to allow for optimal cleaning. In addition, the feeder allows easy chow refilling and can be placed vertically or horizontally in any cage.

The illuminance at the site where mice reside is set by the conventional procedure for dimming LEDs by modifying the duty cycle of the PWM signal powering them, on the basis that pulse-width modulated (PWM) frequency (490 Hz in our case) is much higher than the visual critical flicker frequency (DeRamus and Kraft. 2018). An interesting feature of the setting described here is that in addition to the most common on/off illumination regime employed in rodent research labs (National Research Council, 2011), it also provides an illumination option consisting of a smooth dark-to-light transition and *vice versa* realistically mimicking the natural daily cycle paradigm from sunrise to sunset. This illumination option is of particular interest in investigating the specific dynamics of adaptation to the circadian light cycle (Crowley and Eastman, 2015). Moreover, the setting can use LEDs with different types of white spectra or color light to study the potential effects of the wavelength on circadian adaptation (Dauchy et al., 2019; Kapogiannatou et al., 2016).

To refrigerate the box, we selected an A/C unit based on an easy-to-mount, commercialized module based on the Peltier effect. Although this electrothermal procedure is energetically less efficient than conventional refrigeration by gas compression/expansion, this technology is the most suitable, simple and cheap for our specific application, which requires a relatively low refrigeration power. Indeed, the energy rate necessary for maintaining a refrigerated box at temperature T_box_ when the external temperature is T_ext_ (>T_box_) requires counteracting the heat transfer through the box walls (W’_wall_), the heat involved in changing the temperature of external airflow V′ from T_ext_ to T_box_ (W’_air_), and the heat dissipated by mice metabolism (W’_met_). According to the equation of heat transfer across a flat wall separating two compartments at different temperatures, and taking into account the total surface area and width of the box’s expanded polystyrene foam walls and its thermal conductivity (35 mW·(K·m)^−1^), W’_wall_ ≈ 1.7 W per each °C in the temperature difference T_ext_ − T_box_. W’air can be computed by considering the air density (1.2 g l^−1^) and specific heat capacity (1 J·(g·°C)^−1^), resulting in W’_air_ ≈ 0.4 W per each °C in T_ext_—T_box_. Estimation of W’_met_ is not so straightforward since the dissipation of metabolic heat by mice depends on their ambient temperature (T_box_) (Abreu-Vieira et al., 2015; Kim and Tong, 2017; Gordon, 2017). Whereas at 22°C, the heat dissipation that would correspond to 5 mice is only W’_met_ ≈ 0.8 W (Kim and Tong, 2017), in the case that the ambient temperature (T_box_) decreases by 10°C, W’_met_ can be doubled (Abreu-Vieira et al., 2015). However, even in the worst case, W’_met_ would be negligible compared to W’_wall_ + W’_air_, which would amount to ≈22 W for a temperature difference T_ext_ − T_box_ = 10°C. Hence, the Peltier module we employed (120 W) can provide the necessary refrigeration power, even when considering the relatively low efficiency of this cooling procedure. However, a Peltier module with higher power can be used if required, for instance, to target a relatively low T_box_ (e.g., 5°C) or when using an increased box size. Interestingly, reducing thermal loss can be achieved by using a box with a wider polystyrene wall or by recovering the current one with supplementary polystyrene panels. Of note, a plastic window covered by a removable polystyrene piece can be built into the box lid to periodically observe the animals while keeping the temperature unaffected. We have here focused on the cooling function of the A/C setting since operating the Peltier effect as a heater is much more efficient and poses no potential problems.

We chose a very conservative and safe limit (a maximum of 500 ppm above the lab air level) for the accepted increase in CO_2_ levels within the box. As the metabolic CO_2_ production by five mice (10 ml_CO2_·min^−1^) should equal the difference in CO_2_ content in the air renewal flow V′ at the entrance and outlet of the box (i.e., V' × 5 × 10^−4^ ml_CO2_·ml_air_^−1^), it follows that to keep the 500 ppm maximum increase, the renewal airflow circulated should be V' ≈ 20 l_air_·min^−1^. This flow is 2,000 fold the 5-mice CO_2_ production flow, a figure that would correspond to V' = 500 L min^−1^ for a typical human (250 ml_CO2_·min^−1^ of CO_2_ production), which almost doubles the 5 L s^−1^ (300 L min^−1^) per person recommended for ventilation in buildings (Olesen et al., 2021). In practical terms, it was not necessary to measure V’ because we verified its effectiveness for air renewal by simply measuring the CO_2_ concentration inside the cage with a conventional sensor. The small fan employed had a nominal flow of ≈140 L min^−1^ in open air, which is much higher than required (20 l_air_·min^−1^). However, when subjected to the impedance of the entrance and outlet orifices for air renewal the flow generated by this type of fan (which is not a pressure generator blower) is strongly dampened. As a result, the orifices we set for the air renewal pathway proved to satisfactorily keep safe CO_2_ levels inside the box. Of note, in addition to maintaining an adequate CO_2_ level within the box, the renewal airflow V′ also compensates for the O_2_ consumption by mice metabolism. Given that the flow rate of O_2_ consumption is similar to that of CO_2_ production (respiratory exchange ratio ≈1) (Kim and Tong, 2017), a given V′ would entail a decrease in the O_2_ fraction within the box similar to the corresponding increase in CO_2_ fraction. Hence, assuming a 500 ppm change relative to room air would negligibly reduce the O_2_ concentration in the box from ≈21% to ≈20.95%.

In conclusion, we provide a detailed and free technical description of an easy-to-reproduce device for simultaneously subjecting rodents to different circadian cycles of light, food, and temperature. Among the possible simple changes required to further enlarge the applicability of the device is the use of a larger box to allow a variety of experimental settings. For instance, to host rats or more mice, to provide them with enhanced hosting (Makowska and Weary, 2021; Cait et al., 2022), or to include devices for precisely dosing/weighing food intake (Ali and Kravitz, 2018), monitoring animal movements (Genewsky et al., 2017; Matikainen-Ankney et al., 2019), or to allow animals to exercise (Goh and Ladiges, 2015; Kim et al., 2020).

## Data Availability

The original contributions presented in the study are included in the article/Supplementary Material, further inquiries can be directed to the corresponding author.
