# Supplementary material for: Low-cost, open-source device for simultaneously subjecting rodents to different circadian cycles of light, food, and temperature
Source: Front Physiol. 2024 Feb 16;15:1356787. doi: 10.3389/fphys.2024.1356787 (PMC10904513; doi:10.3389/fphys.2024.1356787)

Low-cost, open-source device for simultaneously subjecting rodents to different circadian cycles of light, food, and temperature

TECHNICAL DESCRIPTION

DISCLAIMER

This document provides technical information on the specific setting and prototype described in the publication indicated above.

Using codes and/or components different from (although similar to) the ones described here may require technical adjustments or adaptations and therefore the final performance of the resulting setting must be specifically assessed.

The authors of this document are not responsible for the use of the information contained herein nor for any setting/device built using such information.

LICENSE

This setting is released under CERN Open Hardware License (OHL) v1.2 and all software and documentation under GNU General Public License (GPL) v3.0.

Content:

1. User instructions
2. Electronic components, circuits and connections
3. Microcontroller (Arduino) code
4. 3D printer drivers

1. User instructions

FRONT PANEL VIEW:

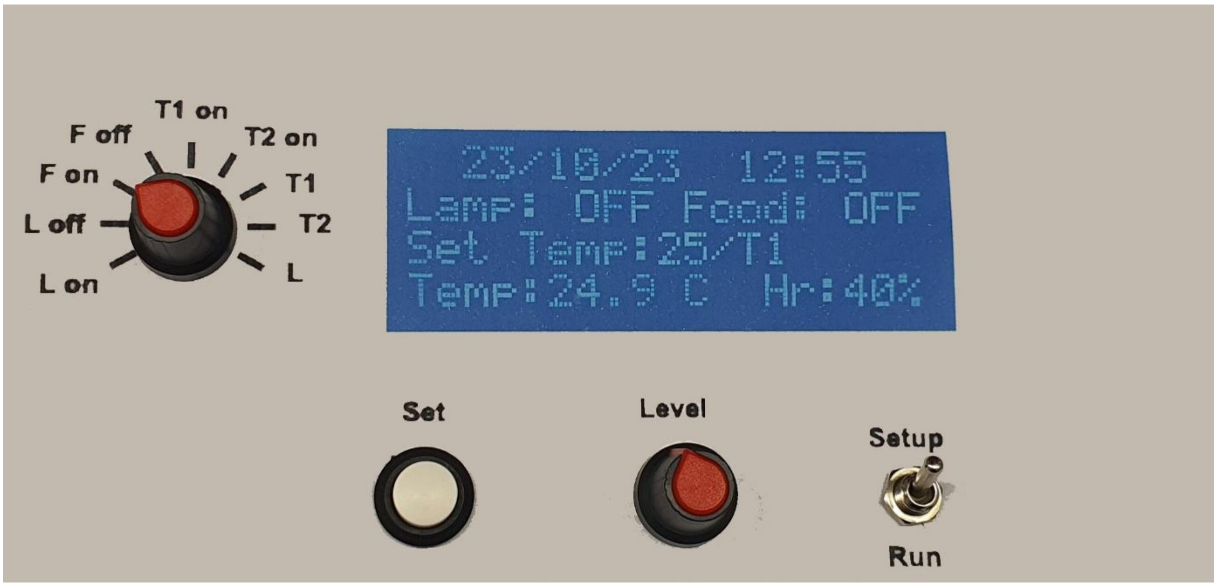

GENERAL DESCRIPTION

The Control Unit has 2 working modes: “Setup” and “Run”.

The “Setup” mode must be selected using the Setup/Run switch on the front panel (bottom right). Once the “Setup” mode is selected, by acting on the rotary switch selector (top left), the different adjustment parameters for cycling temperature, feeding and light we can be selected (as described below).

After setting the parameters, the working mode should be switched to “Run” to normally function according to the established cycling settings.

SETUP mode:

<u>A. Lamp Settings</u>

A.1. “Lamp on” setting. “Lamp on” is the time for the light to switch on.

Put the rotary switch selector on the “Lon” (abbreviation for “Lamp on”) position. The following figure shows the screen in the front panel:

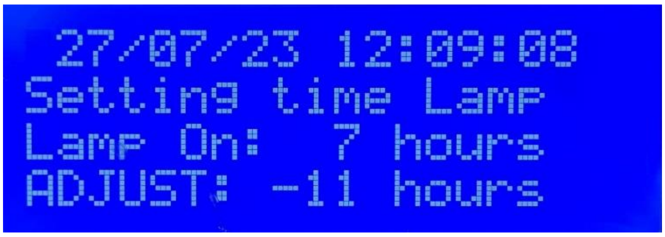

The first line shows the current date and time. The second line shows the setting being adjusted ("Lamp"). The third one shows the "Lamp on" value previously set, and the fourth line shows the value currently being set. This value can be changed by moving the button "Level" in the front panel. The changes applied will appear in real-time on the screen (fourth line). When the desired value appears, the "Set" button (front panel) must be pressed. This finishes the setting of "Lamp on", as will be shown in the third line of the screen.

The adjustment range of the potentiometer in this position ("Lamp on) is between -24 h and 24 h. Setting the time as negative or positive allows for two different time patterns of illumination. When the selected time is positive, the illumination time pattern will be a simple on/off step: constant light during the period of light on and light off otherwise, as shown in the following example for two different light levels (setting of light levels described below in 1.3):

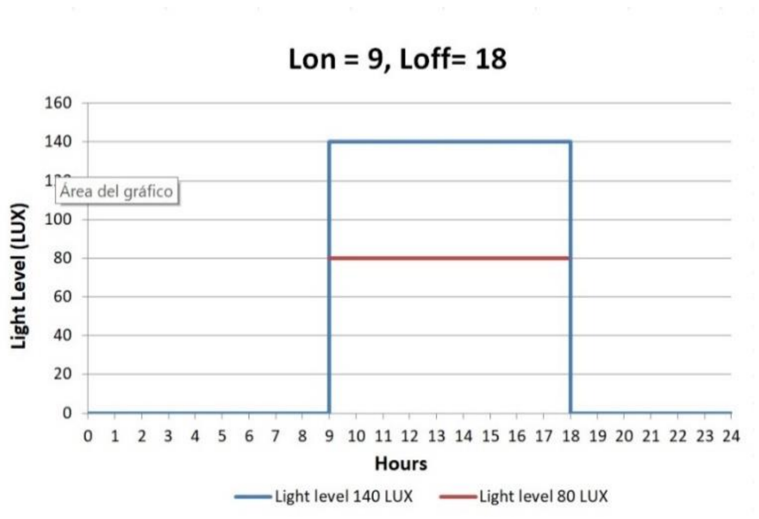

When the selected time is negative, the illumination pattern will follow a progressive increasing/decreasing time course mimicking the solar light intensity, as shown in the following example for two different light values:

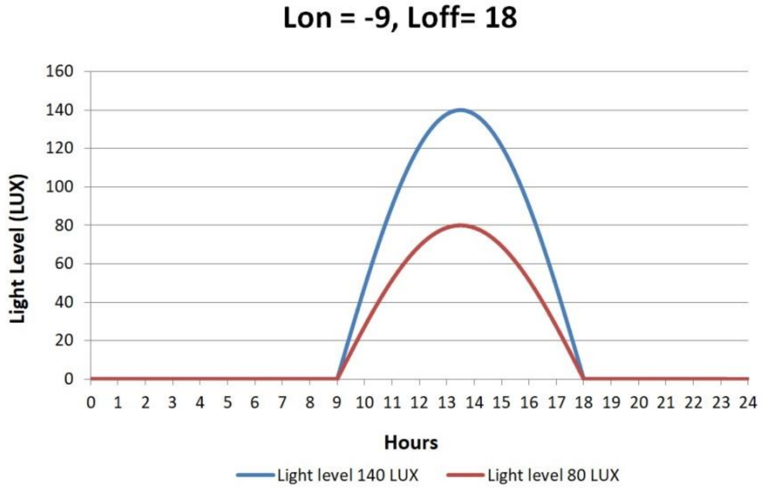

A.2.- “Lamp off” setting. “Lamp off” is the time for the light to switch off.

Put the rotary switch selector on the Loff (abbreviation for “Lamp off”) position:

The following screen appears in the front panel.

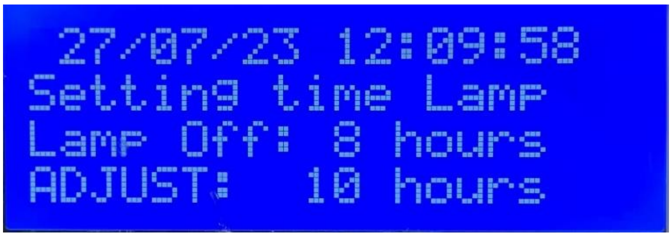

The procedure for setting “Lamp off” is the same as described for “Lamp on”. Here the times allowed by the potentiomer range from 0 to 24 h.

A.3.-Illumination level setting. “Level Max” is the illumination level measured in lux.

Put the rotary switch selector on the L position:

The screen shown is the following:

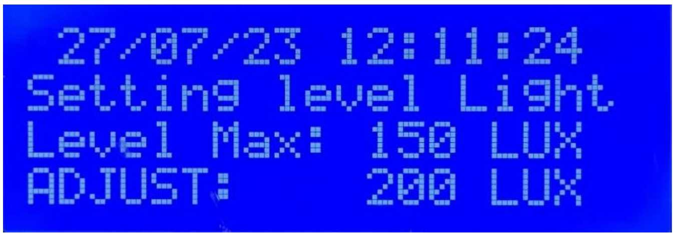

As for all setting procedures, the already set value appears in the third line and the value being adjusted appears in the fourth line. The “Level” button allows modifying the value and the “Set” button stores the value.

Note: The value of L (illumination in Lux) is the constant illumination in the on/off mode, and the peak of illumination when the light pattern mimics the solar light pattern.

The adjustment range of the potentiometer allows values from 14 to 300 Lux.

<u>B. Feeder Settings:</u>

B.1.-“Feed on” setting:

Put the rotary switch selector on the “Fon” (abbreviation for “Feeder on”) position:

“Food On” is the time at which the feeder will allow access to the food. Using the “Level” potentiometer, the time can be selected, and pressing the “Set” button will store the value.

The adjustment range of the potentiometer is between 0 and 24 h.

The screen shown is the following:

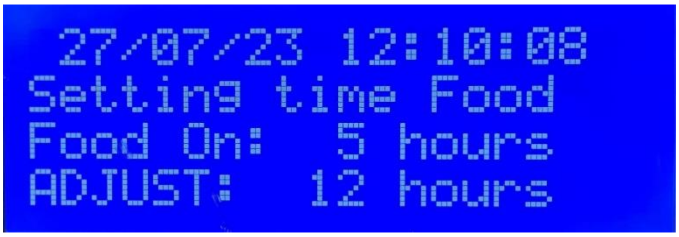

B.2.- “Feed off” setting:

Put the rotary switch selector on the “Foff” (abbreviation for “Feeder off”) position:

“Food Off” is the time at which the feeder will prevent access to the food. Using the “Level” potentiometer the time can be selected and pressing the “Set” button will store the value.

The adjustment range of the potentiometer is between 0 and 24 h

The screen shown is the following:

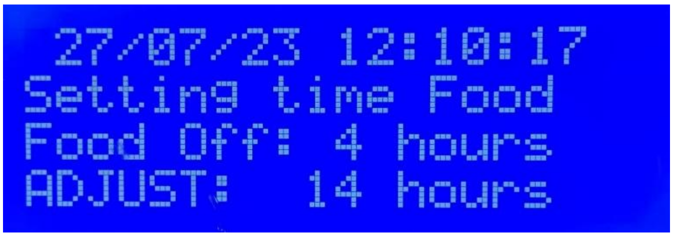

C. Temperature settings:

The system allows to set 2 different constant temperatures per day. A first low value of temperature “T1” starts at time “T1on” and is followed by a high temperature “T2” starting at time “T2on”. For example, T1 and T2 would mimic night and day temperatures, respectively.

C.1. “T1on” setting

Put the rotary switch selector on the “T1on” position:

Use the “Level” and “Set” buttons as previously described to set the “Temp1 On” value.

The adjustment range of the potentiometer is between 0 and 24 hours

The screen shown is the following:

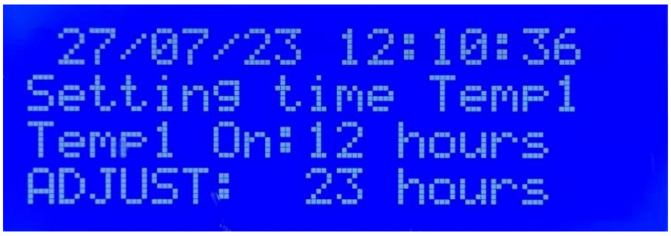

C.2. “T2on” setting

Put the rotary switch selector on the “T2on” position:

Use the “Level” and “Set” buttons as previously described to set the “Temp2 On” value.

The adjustment range of the potentiometer is between 0 and 24 hours

The screen shown is the following:

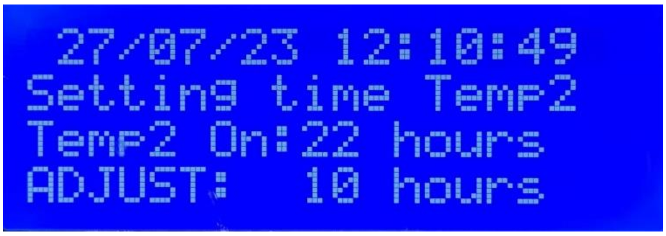

C.3.- “Temp” 1 setting

Put the rotary switch selector on the “T1” position:

Use the “Level” and “Set” buttons as previously described to set the “Temp1” value.

The adjustment range of the potentiometer is between 15 and 36 ºC

The screen shown is the following:

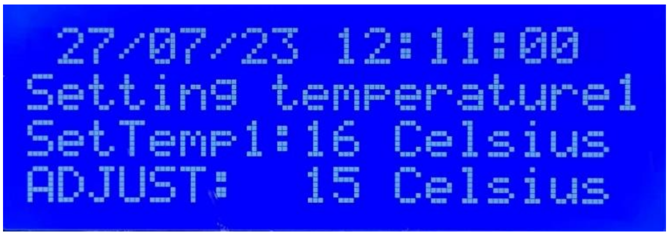

C.4. “Temp” 2 setting

Put the rotary switch selector on the “T2” position:

Use the “Level” and “Set” buttons as previously described to set the “Temp2” value.

The adjustment range of the potentiometer is between 26 and 31 ºC

The screen shown is the following:

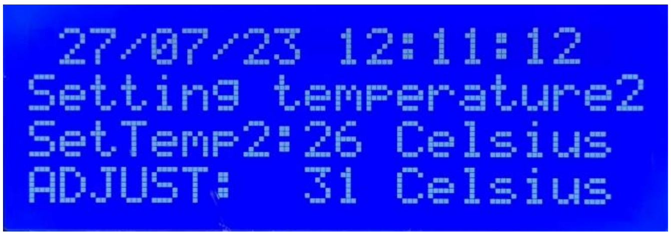

RUN mode

In this mode, the rotary switch selector remains disabled and the system applies the cycling configuration established in “Setup”.

The next figure shows the screen displayed during the “Run” mode.

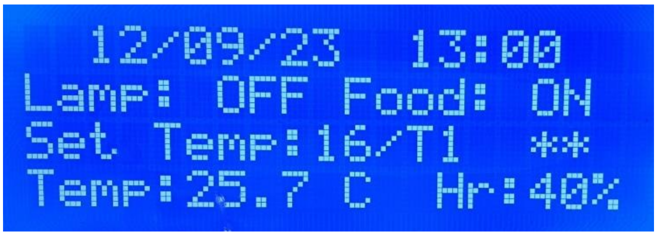

In this example, in addition to the current date and time in the first line, the second line informs that the illuminating lamp is off, that the feeder allows food access to the animals, and that the set temperature of 16 ºC corresponds to T1 (lower temperature). The fourth line shows the real-time measured values of temperature and humidity in the box. The “**” symbol in the fourth line indicates that the A/C system is working. In this example the A/C works because at this moment the set (16ºC) and actual (25.7 ºC) temperatures are different (this situation happens for instance just after the time transitioning from T2 to T1, i.e. from high to low temperature.

At any time during the “Run” mode, pressing “Set” shows the currently set parameters (displayed for 10 seconds on the screen). The next figure shows an example:

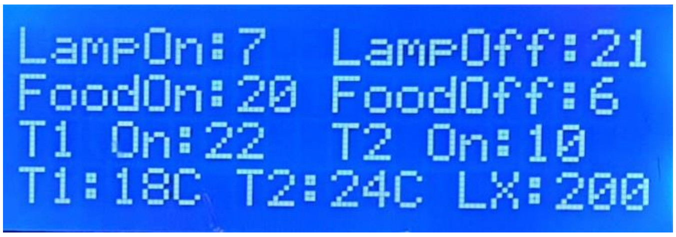

The light is on at 7 h and off at 21 h (daylight). The feeder is on at 20 h and off at 6 h hours (night food availability). A low temperature (T1) of 18 ºC starts at 22 h (night temperature) and a higher temperature (T2) of 24 ºC starts at 10 h (day temperature). The illumination magnitude is set to 200 lux. As “Lamp on”>0, the light time pattern is square-like on/off.

<u>**Note 1**</u>: At any time during operation in the Run mode, the Setup mode can be activated to modify any of the parameters. When switching to “Setup”, the light and feeder will be maintained at the value active when switching to “Setup” and A/C function will be stopped. When switching is resumed to “Run” the system will activate the A/C, light and feeding cycling as required by the current settings.

<u>**Note 2**</u>: For the correct functioning of the Control Unit, it is assumed that the current date and time are adequately set. To this end, please see the comments on section **3._Microcontroller (Arduino) code.**

2. Electronic components, circuits and connections

The prototype has been designed to be affordable and easy to build, providing an open source hardware description to allow free replication, using materials available through e-commerce: I2C temperature sensors (SHT21,SHTC3), 1 module BTS7960 43A Dual H-Bridge, 1 DS1307 AT24C32 Real Time Clock module, Arduino Nano controller with I2C 20x4 digital display and a 2-core 120W Peltier module as well as a 40mm fan). The retail cost of this prototype was less than 300 euros and includes all the electronic circuits and all auxiliary components necessary for its realization, including the power supply. It should be noted that this cost could be considerably reduced by purchasing in bulk. All technical information and detailed circuit schematics (including the optional enclosure via a conventional 3D printer) as well as the driver code needed to build this device are shown in this document and are available in the published Supplementary material files.

The circuit details can be found in the folder "Electronic_circuits" in the "Support files" of the Supplementary material.

The figure shows the internal image of the prototype and the external power source:

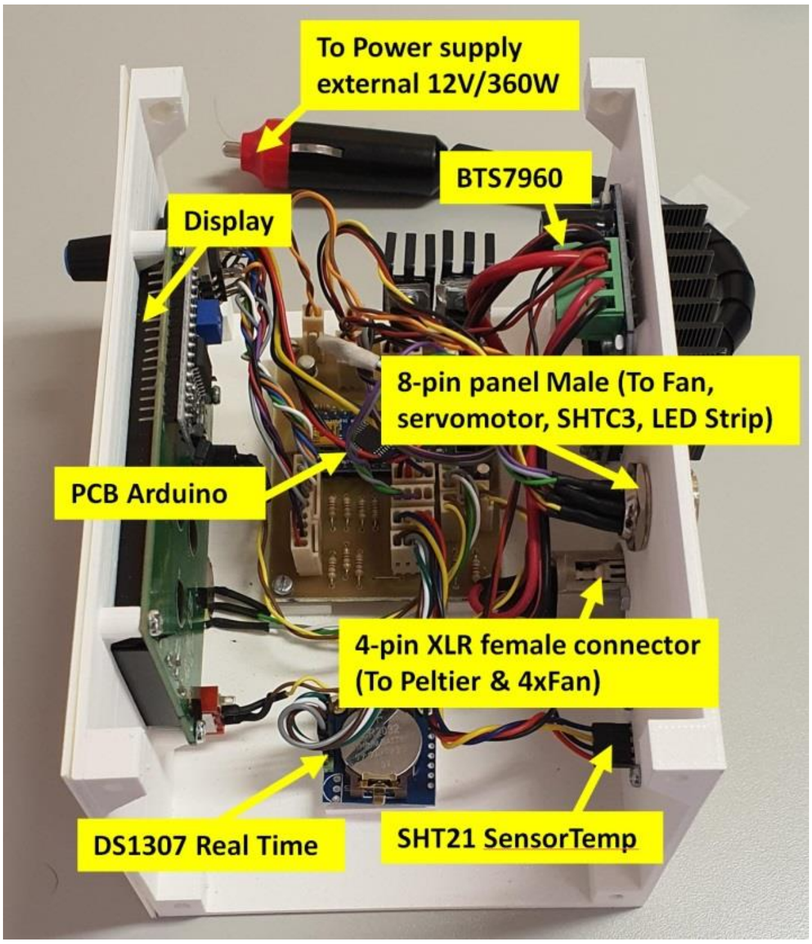

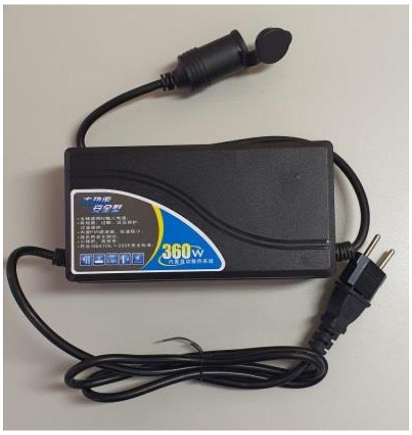

2.1 Schematics of the circuit

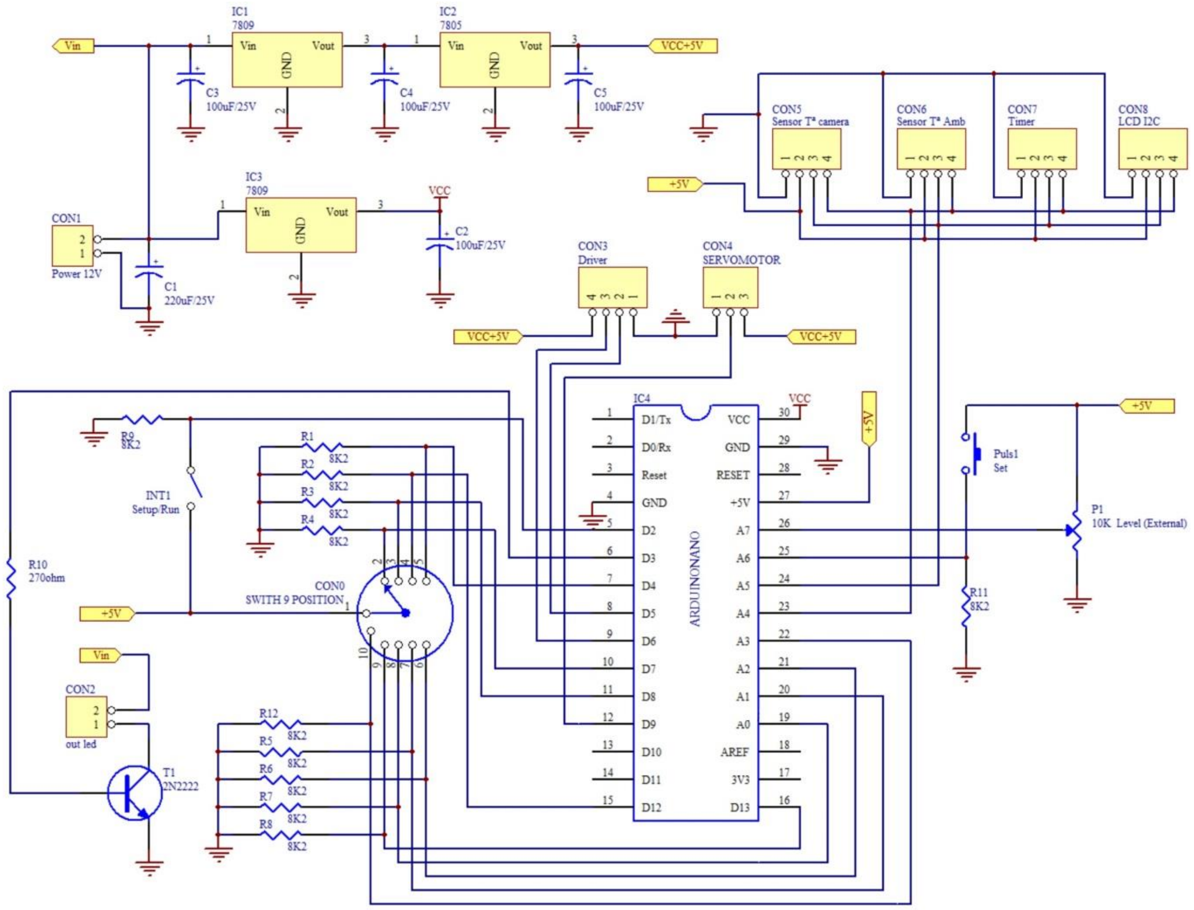

2.2 PCB board layout

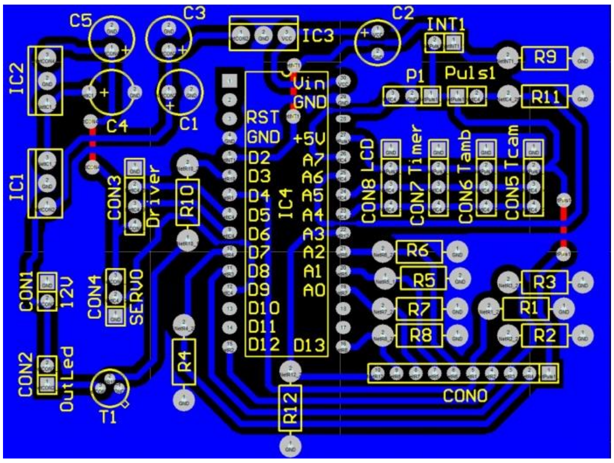

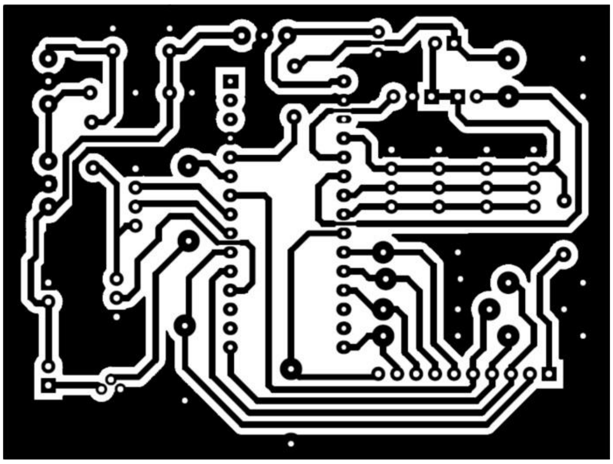

PCB size 60x80 mm

2.3 List of components

PCB Components:

-1x Positive Pre-Sensitized Single-Sided Copper Clad Circuit Board 60x80mm

-Resistors: 10x8,2kΩ, 1x270Ω

-Capacitors 4x100µF (electrolitic), 1x220µF (electrolitic)

-2x MC7809 1Amp voltage regulator

-1x MC7805 1Amp voltage regulator

-1x DIP32 Arduino Nano controller

-1x 2N2222 NPN transistor

-2x TO220 heatsinks, 19x15x10mm (IC1,IC2),

-2x Mica insulator to220

- Connectors: 1x10pin, 5x4pin, 4x2pin, 2x3pin

External components:

-1x SHT21 Ambient temperatura sensor

-1x SHTC3 High Precision Digital Temperature and Humidity Sensor

-1x Hailege BTS7960 43A Dual H-Bridge

-1x RTC I2C DS1307 AT24C32 Real Time Clock Module

-1x I2C LCD 2004 Module, IIC TWI Serial 20x4 Display

-1x Wh148 B10kΩ potentiometer

-1x Push Button 12mm SPST ON/Off

-1x 12 position single pole rotary switch SP12T

-1x toggle switch

-1x Dual core Peltier thermoelectric cooling 12V/120W

-1x 12V LED Strip

-1x AAB cooling Fan 4 (5,8 m3/h)

-1x Male aerial cigarette lighter connector

-1x 4-pin XLR male aerial connector

-1x 4-pin XLR female panel connector

-1x 8-pin panel Male connector gx16

-1x 8-pin aerial female connector gx16

<u>Feeder components:</u>

-1x Servo Motor Hitec HS322

-1x 1000ml Stainless Steel Water Bottle

-5x Solid Stainless Steel Round Rods 2.5mm x 300mm

-1x Stainless Steel Thin Sheet Sheet 0.1mm

<u>Other components</u>

-1x Power supply external 12V/360W

-1x Expansion box   59,5x39,5x40cm

-PLA printer filament (550g)

2.4 Conexions

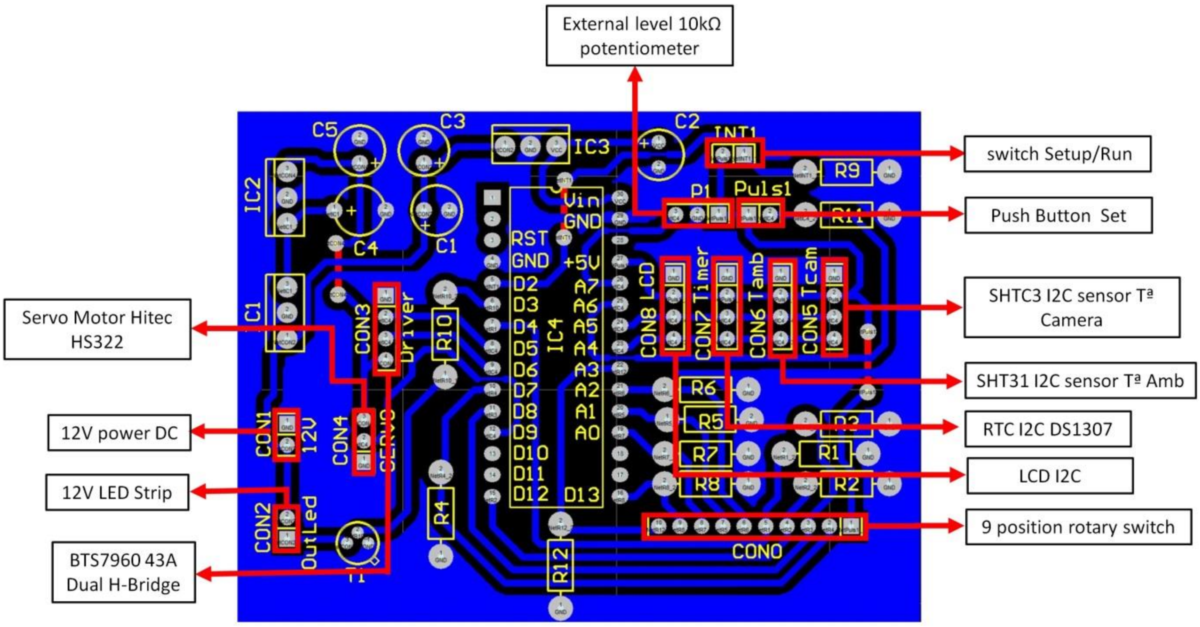

- **Position rotary switch connections**

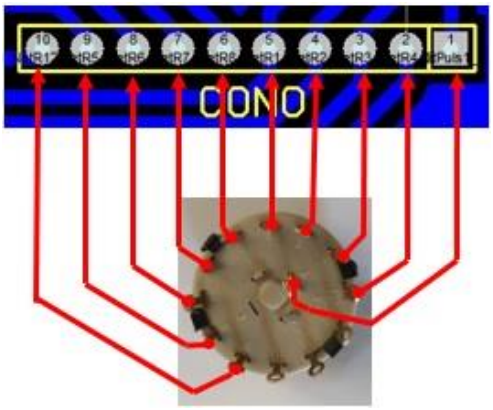

- **Connections I2C temperature, clock and LCD display modules**

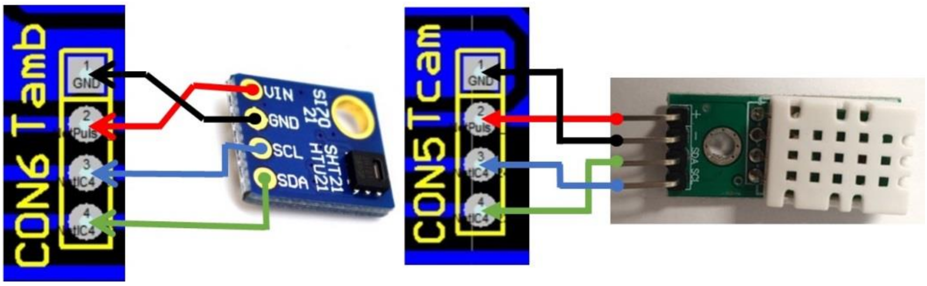

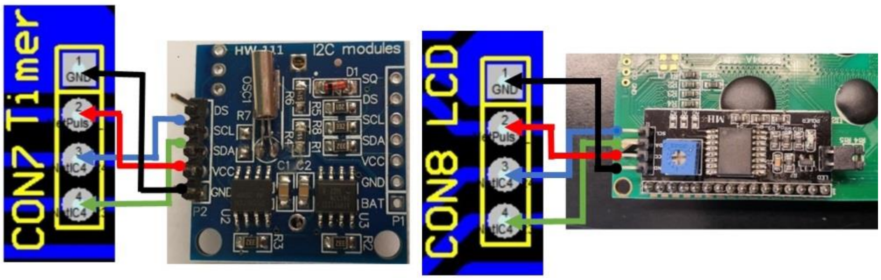

- **Connection SETUP/RUN switch, level potentiometer and Set button**

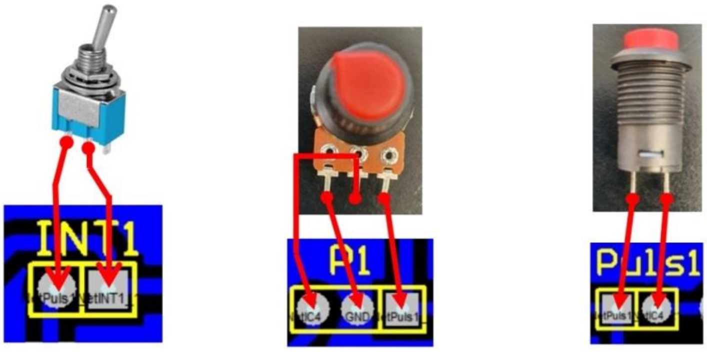

- **Hitec servomotor connection**

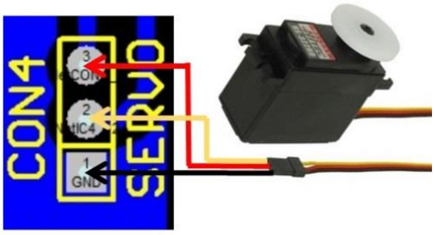

- **Hailege BTS7960 driver connection**

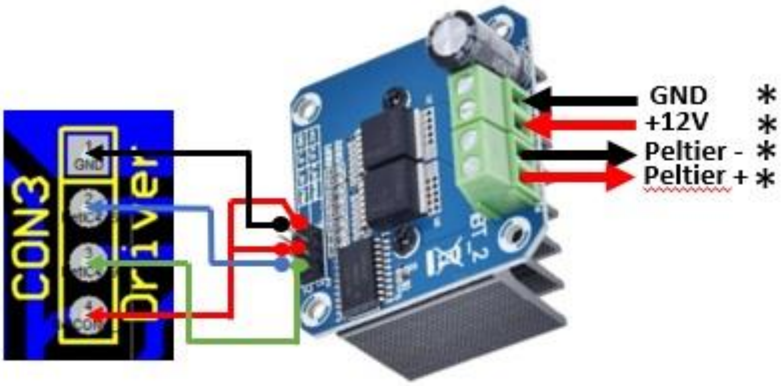

* minimum cable section 1.5 mm2

- Peltier conection

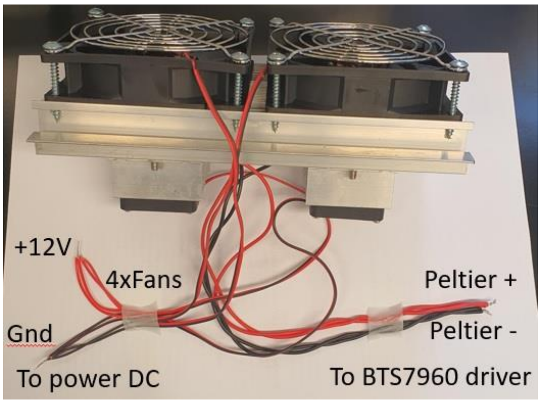

- **air renewal fan**

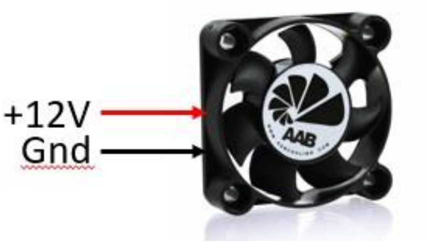

- **led bar (15 leds)**

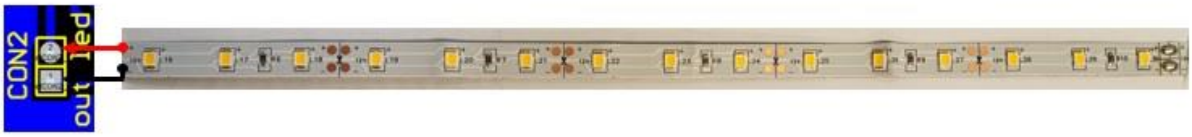

3. Microcontroller (Arduino) code

The Arduino codes can be found in the folder "Arduino" in the "Support files" of the Supplementary material:

The "Arduino_code" folder contains the code for running the device.

The "Arduino_setting_clock" folder contains de file to initially set the time and date of the internal clock of the device.

4. 3D printer drivers

4.1. Control unit box

The 3D printer driver files can be found in the folder "3D_Print_Control_envelope" in the "Support files" of the Supplementary material.

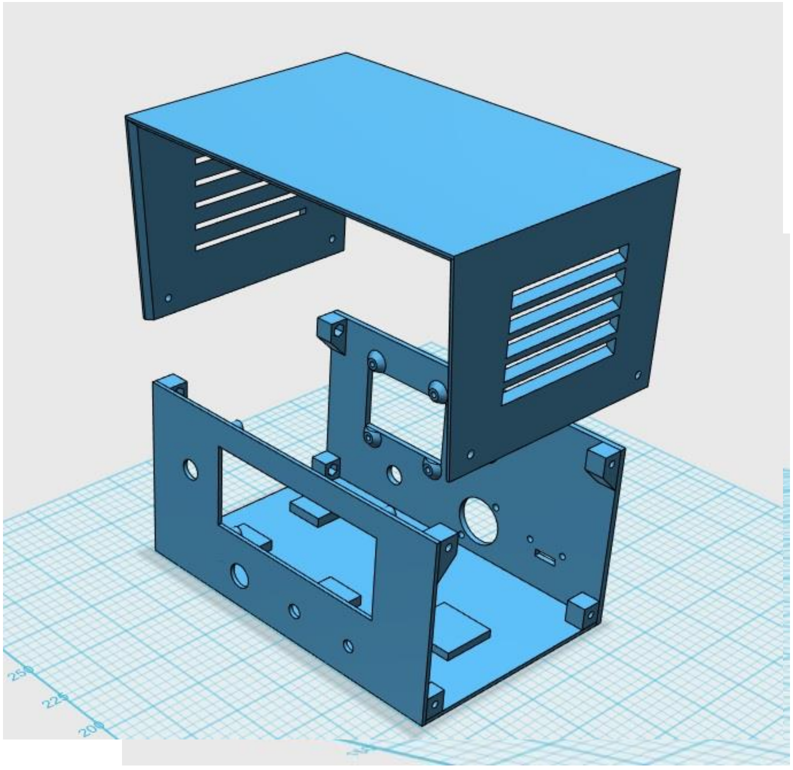

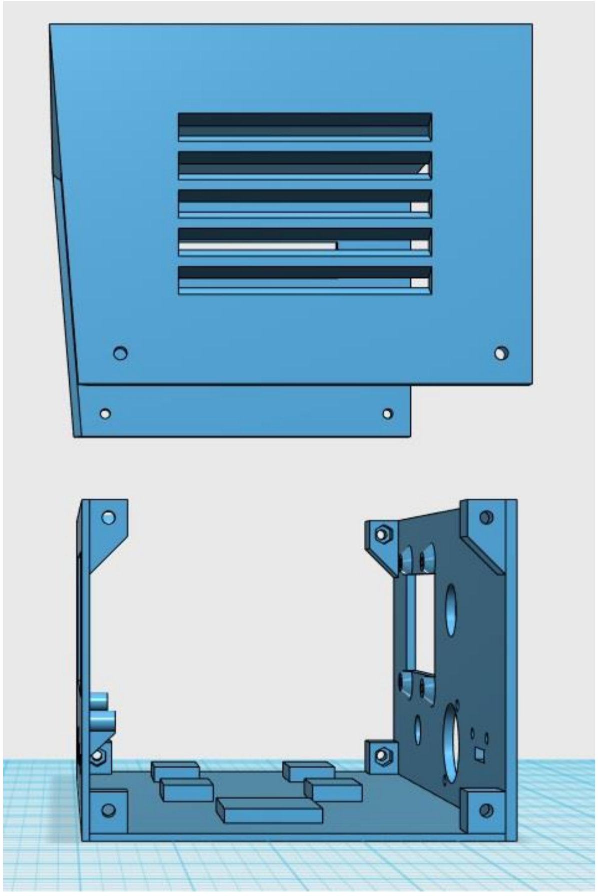

- Ambient temperature sensor

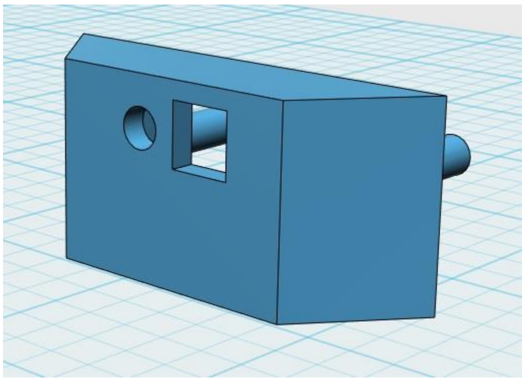

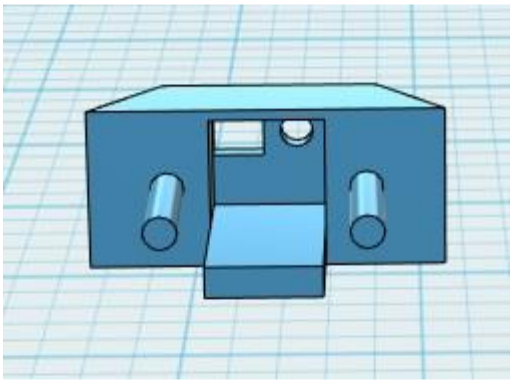

4.2. Adapters for the thermal box

The 3D printer driver files can be found in the folder "3D_Print_Complements_Thermal_Box" in the "Support files" of the Supplementary material.

-Peltier adapter

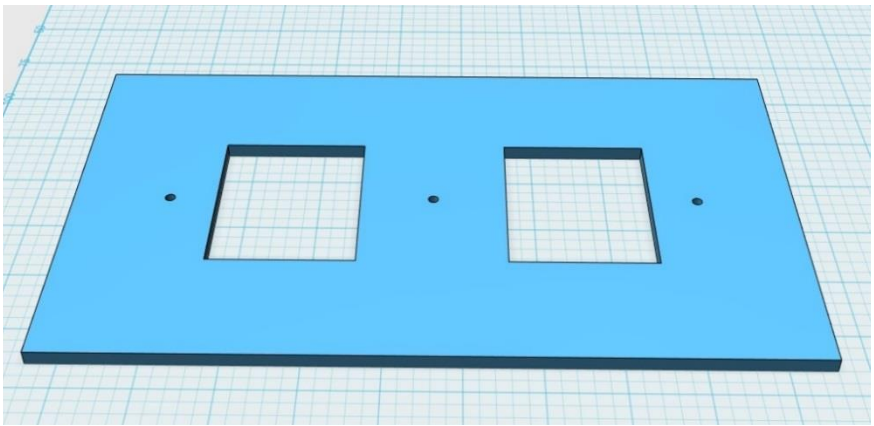

-air renewal fan

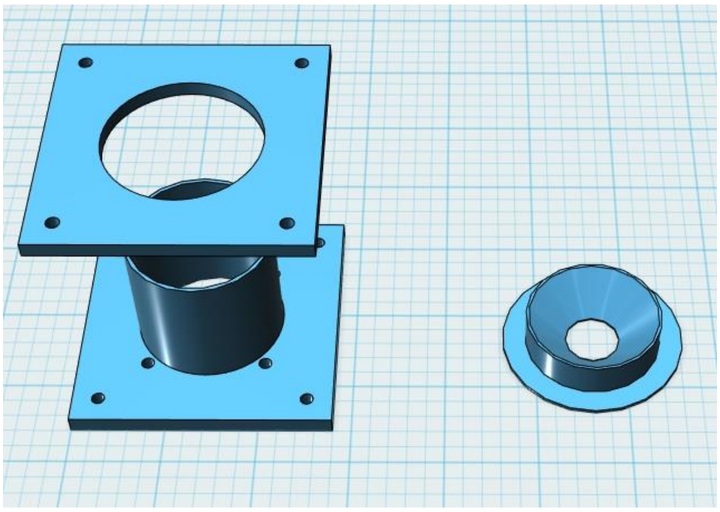

-cable gland

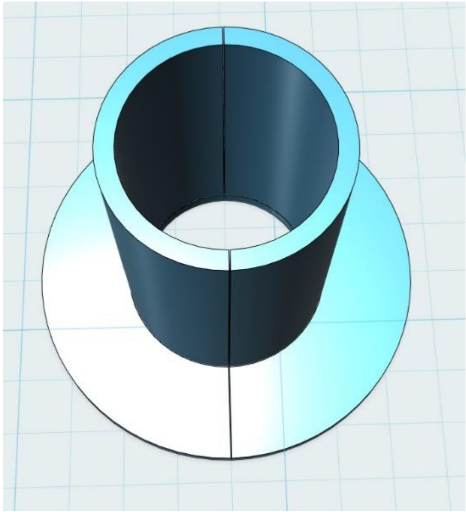

- 2 x LED strip

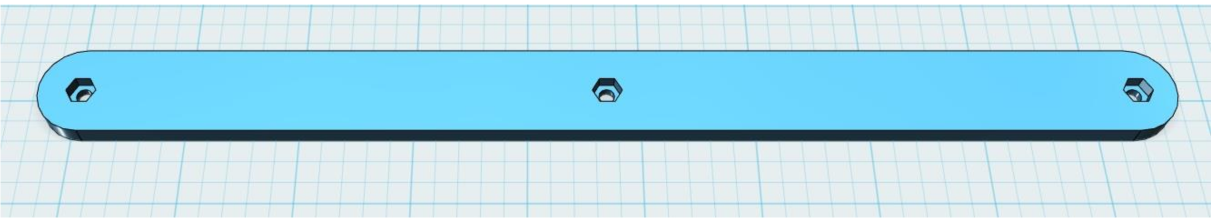

4.3. Feeder parts

The 3D printer driver files can be found in the folder "3D_Print_Feeder" in the "Support files" of the Supplementary material.

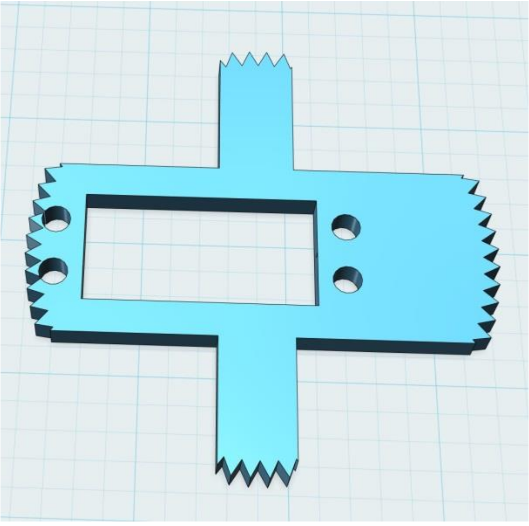

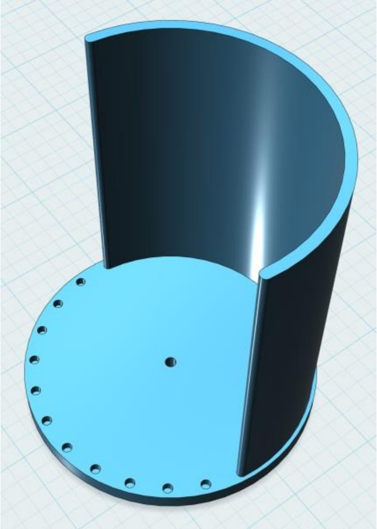

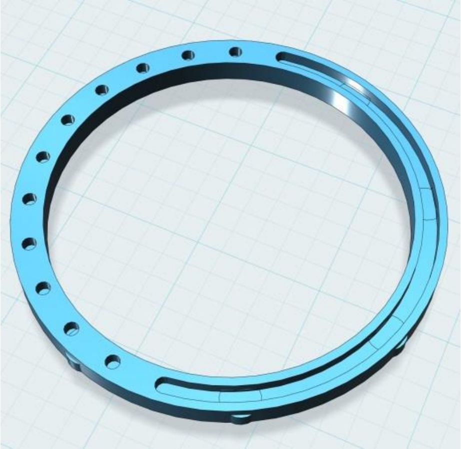

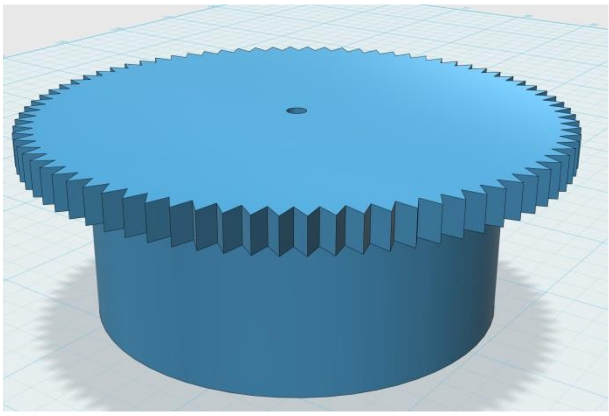

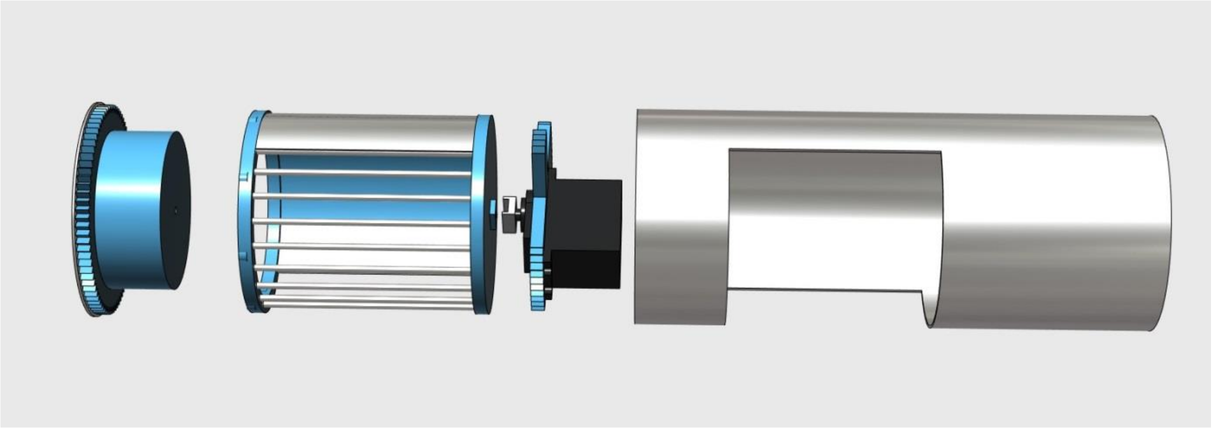

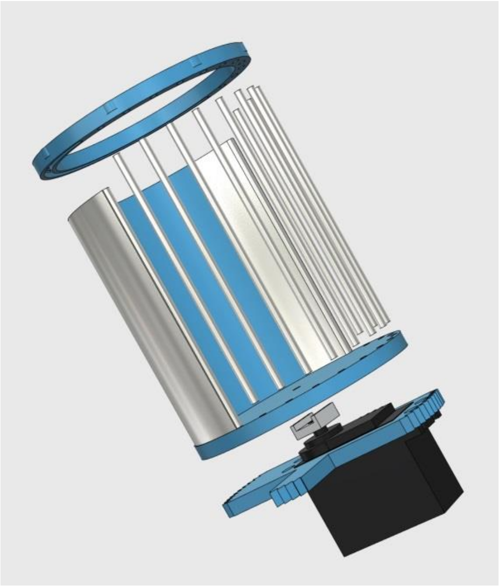

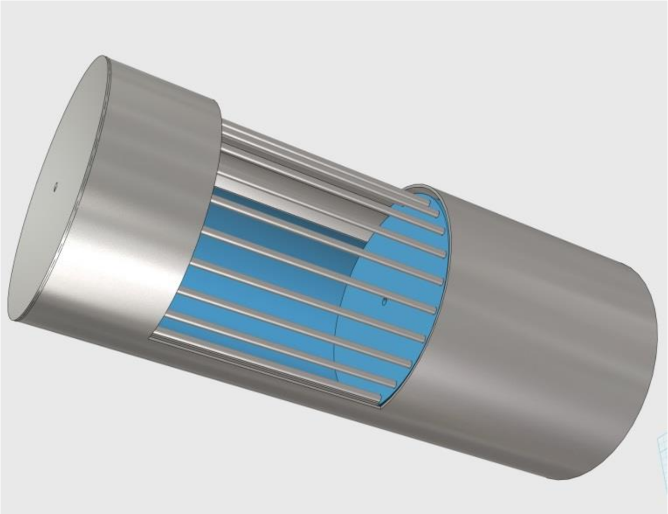

Supplement: Supplementary file 1 [file DataSheet1.ZIP › Supplementary_material/Technical_Description-Supplementary.pdf]
